# CXCR7 Protein Expression in Human Adult Brain and Differentiated Neurons

**DOI:** 10.1371/journal.pone.0020680

**Published:** 2011-05-31

**Authors:** Saori Shimizu, Michael Brown, Rajarshi Sengupta, Mark E. Penfold, Olimpia Meucci

**Affiliations:** 1 Department of Pharmacology and Physiology, Drexel University College of Medicine, Philadelphia, Pennsylvania, United States of America; 2 Department of Microbiology and Immunology, Drexel University College of Medicine, Philadelphia, Pennsylvania, United States of America; 3 ChemoCentryx, Mountain View, California, United States of America; Brigham and Women's Hospital, Harvard Medical School, United States of America

## Abstract

**Background:**

CXCR7 and CXCR4 are receptors for the chemokine CXCL12, which is involved in essential functions of the immune and nervous systems. Although *CXCR7* transcripts are widely expressed throughout the central nervous system, little is known about its protein distribution and function in the adult brain. To evaluate its potential involvement in CXCL12/CXCR4 signaling in differentiated neurons, we studied CXCR7 protein expression in human brain and cultured neurons.

**Methodology/Principal Findings:**

Immunohistochemistry and RT-PCR analyses of cortex and hippocampus from control and HIV-positive subjects provided the first evidence of CXCR7 protein expression in human adult neurons, under normal and pathological conditions. Furthermore, confocal microscopy and binding assays in cultured neurons show that CXCR7 protein is mainly located into cytoplasm, while little to no protein expression is found on neuronal plasma membrane. Interestingly, specific CXCR7 ligands that inhibit CXCL12 binding to CXCR7 do not alter CXCR4-activated survival signaling (pERK/pAkt) in rat cortical neurons. Neuronal CXCR7 co-localizes to some extent with the endoplasmic reticulum marker ERp29, but not with early/late endosome markers. Additionally, large areas of overlap are detected in the intracellular pattern of CXCR7 and CXCR4 expression.

**Conclusions/Significance:**

Overall, these results implicate CXCR4 as the main CXCL12 signaling receptor on the surface of differentiated neurons and suggest that CXCR7 may interact with CXCR4 at the intracellular level, possibly affecting CXCR4 trafficking and/or coupling to other proteins.

## Introduction

CXCR7, formerly known as RDC1, is a recently identified binding partner for the chemokine CXCL12 (also known as SDF-1), which primarily signals via its specific receptor CXCR4 [1]–[3]. CXCR7 transcripts are widely and highly expressed in many organs, including the brain [2], [4]–[6]. However, discrepancy between RNA levels and surface expression of CXCR7 has been noted in various organs [2] suggesting that regulation of CXCR7 expression is tightly regulated at the protein level and may be tissue-specific.

As with other chemokine receptors, CXCR7 belongs to the superfamily of seven-transmembrane (7TM) G protein coupled receptors, though current evidence indicates that CXCR7 does not stimulate typical G protein-dependent pathways, and may act as a β -arrestin-biased receptor [7]–[9] and/or as a chemokine scavenger, particularly during development or in the tumor microenvironment – i.e. sequestration of CXCL12 by binding to CXCR7 may help create a CXCL12 gradient in the extracellular space, resulting in proper chemotaxis/migration of CXCR4-expressing cells [8], [10], [11]. Other studies suggest that CXCR7 may form heterodimers with CXCR4 [9], [12]–[15], thus serving as a modulator of CXCR4 signaling, but the functional outcome and mechanistic insights are still controversial. Enhanced responses to CXCL12 (i.e. calcium flux and mitogen-activated protein kinase activation) were observed in CXCR4 positive cells expressing recombinant CXCR7 as compared to cells that express only the endogenous CXCR4 protein [13]. On the other hand, variable results have been reported regarding the potential role of CXCR7 in modulating CXCL12-induced chemotaxis [14] though some controversy still exists about the endogenous expression of CXCR7 in lymphocytes. Also, CXCR7 can interfere with CXCR4-dependent rapid integrin activation [15] and transendothelial migration [12]. Overall, these results suggest that CXCR7 can modulate CXCL12/CXCR4 signaling in non-neuronal cells via multiple mechanisms.

The CXCL12/CXCR4 axis is essential to proper brain development and is involved in homeostasis of the mature brain, as it regulates fundamental neuronal and glial functions (i.e. cell survival, differentiation, migration, and synaptic activity) [16]–[18]. Furthermore, this chemokine/receptor pair is implicated in various neuropathologies and neuroinflammatory conditions, including brain tumors and neuroAIDS [16], [19]–[22]. Interestingly, CXCR7 may also support HIV-1 infection in glioma cells under specific circumstances [23]. Hence, it is important to examine CXCR7 protein expression and function in normal and disease states in order to determine its ability to regulate CXCL12-stimulated signaling in the brain and influence neuropathology.

To this end, we studied the expression of CXCR7 in cultured neurons and in the brains of HIV-1 positive and negative subjects. Our data provide the first evidence of CXCR7 protein expression in normal and diseased adult human neurons *in situ* and show that, in differentiated cultured neurons, CXCR7 is predominantly expressed in intracellular compartments, it is virtually absent from the cell surface, and does not alter CXCR4-mediated survival signaling through pERK/Akt. Interestingly, neuronal CXCR7 largely co-localizes with the intracellular pool of CXCR4, suggesting that it may interact with CXCR4. Along with previous evidence, these results underscore the role of the CXCL12/CXCR4 axis in cortical neurons and indicate that CXCR7 protein in these cells is tightly controlled both temporally and spatially, and it could contribute to the intracellular fate of CXCR4.

## Materials and Methods

### Ethics statement

All experiments involving animals were performed according to protocols approved by the Institutional Animal Care and Use Committee (Protocol Number: 18547) at Drexel University College of Medicine. This study used archived human brain tissue samples from HIV patients and control individuals. The samples have been provided by brain banks participating to the National NeuroAIDS Consortium and included specimens from unidentified patients of both sexes and different races. The Institutional Review Board of Drexel University has reviewed our protocol and approved it as Exempt Research (#18452).

### Reagents

The anti-CXCR7 (clone11G8) antibody, which recognizes the first 20 residues of the N-terminus of CXCR7, and the high-affinity CXCR7 ligands were a gift from ChemoCentryx [2], [12], [24], [25] and used as reported below. The small-molecule compounds tested in the present study include: CCX733, CCX754, CCX771, and CCX704 (an “inactive” analogue of CCX771). The polyclonal anti-CXCR7 antibody, ab72100, was purchased from Abcam (Cambridge, MA). Unless otherwise specified, reagents and media for the cell culture were purchased from Invitrogen/Gibco (Carlsbad, CA) or Sigma-Aldrich (St. Louis, MO).

### Immunohistochemistry and source of human tissue

Immunohistochemistry analysis of human brain tissue was conducted as we previously described [26]. A Breast Cancer Tissue Array (BR721, US Biomax, Rockville, MD) containing normal and 24 cancer samples was used to confirm CXCR7 antibody specificity in our hands, based on previous reports [2], [27]. A human organ tissue array containing 33 types of normal organs was purchased from US Biomax (FDA996t). A total of nine brain autopsy samples from two groups of patients (Table 1) were obtained from the tissue banks of the National NeuroAIDS Tissue Consortium (NNTC) [28]. Specimens included samples from the cerebral cortex and hippocampus of patients with HIV infection or control patients (HIV negative). After deparaffinization and quenching of endogenous horseradish peroxidase, sections were incubated at 95°C in Target Retrieval Solution (Dako, Carpinteria, CA) for 1 h. Endogenous avidin/biotin was blocked in blocking buffer (Vector Laboratories, Burlingame, CA). Sections were incubated in Tris-buffered saline with 10% normal donkey serum (Jackson ImmunoResearch, West Grove, PA), 2% milk, and 2% bovine serum albumin for an additional hour, and then overnight with an anti-CXCR7 monoclonal (10 µg/mL, 11G8, ChemoCentryx) or an anti-CXCR7 polyclonal antibody (10 µg/mL, ab72100, Abcam). Comparison between these two antibodies for CXCR7 showed similar results in adjacent sections, as shown later; however, 11G8 was preferred in the present study, due to its high specificity in both tissue preparations and isolated cells [24]. Biotin-conjugated donkey anti-mouse or a biotin-conjugated donkey anti-rabbit antibody (1∶500, Jackson ImmunoResearch) was used to detect primary antibodies. Staining was visualized with 3,3′-diaminobenzidine (Vector Laboratories). Serial sections of tissue were stained with hematoxylin and eosin (H&E). Negative controls (i.e., isotype control or no primary Ab) were included as indicated. Sections were observed under a Nikon microscope (OPTIPHOT-2) connected to a CCD camera (DP70, Olympus, Melville, NY) or Zeiss Axio microscope connected to a CCD camera (Nuance 2.10, CRi, Woburn, MA), and images were taken using the DP (Olympus) or Nuance software, respectively. For identification of CXCR7 positive neurons, sections were incubated over night with monoclonal anti-CXCR7 and polyclonal MAP2 (a+b) (Millipore), followed by 30 minutes incubation with Alexa 647–conjugated goat anti-mouse and Alexa 546–conjugated goat anti-rabbit antibodies (1∶200, Invitrogen), respectively. Images were captured with a Zeiss LSM5 Exciter laser scanning confocal microscope (Thornwood, NY) using sequential imaging to prevent interchannel cross-excitation between fluorochromes.

**Table 1:** Patient demographics, viral load, and CD4 counts.

Group	Age	Sex	Race	PMI (h)	Area	CD4 count (cells/mm^3^)	Plasma viral load (copies/mL)	CSF viral load (copies/mL)
Control	52	Male	White	17.5	C/H	N/A	N/A	N/A
Control	59	Female	Black	10	C/H	N/A	N/A	N/A
Control	59	Male	White	18	C/H	N/A	N/A	N/A
HIV	64	Male	White	5.5	C/H	61	75	7484
HIV	44	Male	Black	21.3	C/H	234	16,909	618
HIV	44	Male	White	35	C/H	147	<50	N/A
HIV	30	Male	Black	6	C/H	8	104,300	14
HIV	43	Male	Hispanic	31	C/H	3	110,493	65
HIV	43	Male	Black	6	C/H	10	48,520	134

PMI: post-mortem interval, h: hour, C: cortex, H: hippocampus.

### Cell Cultures

Human neurons were purchased from ScienCell Research Laboratories (San Diego, CA), maintained in the neuronal culture medium as indicated by the vendor, and used at 14–21 days *in vitro* (DIV). Rat cortical neurons were obtained from rat embryos (E17) and cultured in the absence of glia in serum-free defined media, as we previously described [29], [30]; cells were typically used for experiments in their second week of culture (8–14 DIV), i.e. when they are fully differentiated. MAP2 and GFAP staining were used to identify neurons and astroglia, respectively. Human breast cancer cells (MDA-MB-435) and human glioblastoma cells (U87MG, U343MG) were cultured as previously described [2], [15], [31], [32].

### Immunocytochemistry

Cells on coverslips were labeled with the Image-iT LIVE Plasma Membrane and Nuclear Labeling kit from Invitrogen/Molecular Probes and then fixed in 4% paraformaldehyde. Cells were then permeabilized with 0.1% Triton X-100 for 5 minutes. The following antibodies were used: mouse anti-CXCR7 (50 µg/mL, ChemoCentryx; 11G8), mouse anti-CXCR4 (1∶200, R&D Systems, Minneapolis, MN; 12G5), rabbit anti-MAP2 (a+b) (1∶200; Millipore, Billerica, MA), rabbit anti-GFAP (1∶200; Santa Cruz biotechnology, Santa Cruz, CA), rabbit anti-EEA1 (1∶200; Cell Signaling), rabbit anti-Rab7 (1∶50; Cell Signaling), rabbit anti-Rab11 (1∶100; Cell Signaling), and rabbit anti-ERp29 (1∶500; Cell Signaling). Double staining was performed sequentially with CXCR7 (Alexa 488–conjugated donkey anti-mouse as a secondary antibody) always preceding the other primary antibodies, followed by biotin-conjugated antibodies (either anti-mouse or anti-rabbit) as secondary antibodies, and visualized by streptavidin conjugated to Alexa Fluor 647. Confocal images were captured with either a Leica TCS SP2 (Bannockburn, IL) or a Zeiss LSM5 Exciter laser scanning confocal microscope (Thornwood, NY) using sequential imaging to prevent interchannel cross-excitation between fluorochromes. Images were captured every 0.4–0.5 µm for *z*-scan.

### RT-PCR

Total RNA was extracted using the RNeasy mini kit (Qiagen, Valencia, CA). RNA obtained from rat and human cell lines was treated with DNase prior to RT reaction. cDNA was synthesized from total RNA. The following primers were used for amplification: rat CXCR7: 5′-GTGCAGCATAACCAGTGGCC-‘ and 5′-AGCAAAACCCAAGATGACGGA-3′ [33], human CXCR7: 5′-AGCACAGCCAGGAAGGCGAG-3′ and 5′-TCATAGCCTGTGGTCTTGGC-3′, human 28S: 5′-GTTCACCCACTAATAGGGAACGTGA-3′ and 5′-GGATTCTGACTTAGA GGCGTTCAGT-3′, human 5′- GAPDH: CATTGACCTCAACTACATGG-3′ and 5′-GGGCCA TCCACAGTCTTCTG-3′, rat aldolase A: 5′-AACCAATGGCGAGACCACTAC-3′ and 5′-AATTTCAGGCTCCACAATGG-3′. PCR was performed as follows: 4 minutes at 95°C, followed by 35 cycles of 30 s at 95°C, 30 s at 61°C, and 60 s at 72°C. For gel electrophoresis analysis, each amplicon was generated and visualized using ethidium bromide staining after electrophoresis on a 2% agarose gel. *Aldolase A* (rat), *28S* (human) and *GAPDH* (human) were used as loading controls.

### Western Blot

Cells were washed with ice-cold balanced salt solution and scraped in lysis buffer (50 mM Tris, 150 mM NaCl, 0.5% sodium deoxycholate, 0.1% sodium dodecyl sulfate [SDS], 10 mM Na_4_P_2_O_7_, 5 mM ethylenediaminetetra-acetic acid [EDTA], 1% Triton X-100, 1 mM dithiothreitol, protease and phosphatase inhibitor cocktails) as reported previously [34]–[36]. Equal amounts of proteins as determined by the bicinchoninic acid assay from Thermo Scientific (Rockford, IL) were loaded in each lane, separated by sodium dodecyl sulfate–polyacrylamide gel electrophoresis (SDS-PAGE), and transferred to polyvinylidene fluoride membranes for immunoblotting. The following primary antibodies were used for ERK/Akt studies as previously reported [30]: anti-pERK1/2 (1∶1000, cat#9101, Cell Signaling, Danvers, MA), anti-ERK1/2 (1∶1000, cat#9102, Cell Signaling), anti-pAkt (1∶1000, cat#9271, Cell Signaling), anti-Akt (1∶1000, cat#9272 Cell Signaling). Anti-actin was used for loading control (1∶5000, cat#A2066, Sigma-Aldrich). Different monoclonal and polyclonal antibodies against CXCR7 were tested, often with inconclusive results (not shown). The polyclonal antibody from Abcam ab72100 (1∶500) is shown here; however, since this antibody can produce non specific bands in non-neuronal cells [24], additional experimental approaches were used to confirm expression of CXCR7 in rat neurons, as described in the results. Bands were detected by chemiluminescence using SuperSignal West Femto (Thermo Scientific), and analyzed using the FluorChem 8900 apparatus from Alpha Innotech (San Leandro, CA). Cell surface protein separation was performed as we previously reported [30] using a thiol-cleavable amine-reactive biotinylation reagent (Thermo Scientific). After isolation of biotin-labeled cells, SDS-PAGE and Western blotting were performed.

### Binding Assay

Cells (2×10^5^ per well) were diluted in HBSS containing 0.1% BSA and transferred to 96-well plates (100 µl per well) containing 5 µl per well recombinant CXCL11 or CXCL12 (final concentration 100 nM) or compounds CCX771, CCX704 or AMD3100 (final concentration 1 µM). One hundred µl per well of cold buffer containing 0.025 µCi (1 nM) 125I-CXCL12 (PerkinElmer) was added and the plates were shaken at 4°C for 3 h. Cells were transferred onto polyethyleneimine-treated GF/B glass fiber filters (PerkinElmer) with a cell harvester (Tomtech) and washed with 25 mM HEPES, 500 mM NaCl, 1 mM CaCl2, 5 mM MgCl2, pH 7.1. Fifty µl per well of MicroScint-20 (PerkinElmer) was added to the filters and cpm were measured on a Packard TopCount scintillation counter (PerkinElmer). Assays were performed in triplicate.

### Statistical Analysis

One-way analysis of variance, followed by the Newman-Keuls multiple comparison procedure, was used for statistical analysis. All data are reported as the mean ± SEM (n = 3 for Western blots and n = 4 for radioligand binding assays).

## Results

### CXCR7 Expression in Adult Human Neurons: analysis of normal and HIV-positive brains

**Figure 1:**
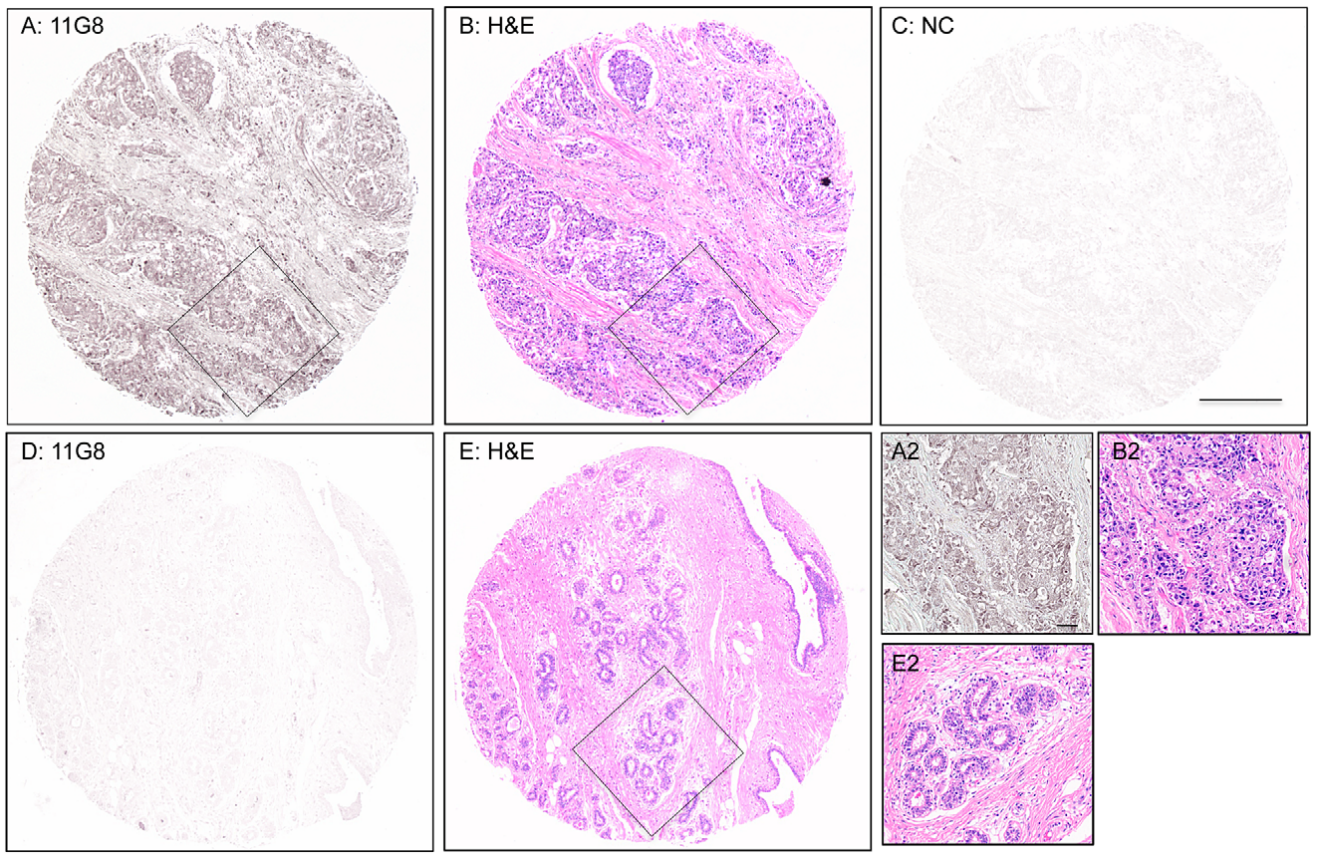
Staining of breast carcinoma with the CXCR7 antibody 11G8. A tissue array, containing samples from infiltrating ductal carcinoma and normal breast tissue, was stained to examine CXCR7 expression using the monoclonal antibody 11G8 (10 µg/mL)-as described in the methods. Representative images from infiltrating ductal carcinoma or normal tissue are shown here. Adjacent sections of the same samples were stained with Hematoxylin and Eosin (H&E) or the isotype (i.e. mouse IgG1) control, as indicated for each panel. (A/B/C) Infiltrating ductal carcinoma (NC = isotype control). (D/E) Normal breast tissue. (A2, B2, E2) High magnification of black squares in A, B, and E, respectively. Scale bars: 400 µm (A–E), 100 µm (A2, B2, E2).

Previous studies have demonstrated that CXCR7 protein is specifically expressed in human breast cancer as opposed to normal breast tissue [27]. Therefore, our first step consisted in testing our staining protocol with the 11G8 antibody using a human tissue microarray that contained normal breast tissue and multiple breast carcinomas on the same slide. As expected, CXCR7 was expressed in most of the carcinomas but not in normal breast tissue. Two examples are reported in Figure 1 (A/D). This figure also shows the typical alterations of the glandular structure in the cancer tissue as compared to normal (counterstaining in 1B/E), higher magnification images of selected areas (square insets), and a negative control (1C).

**Figure 2:**
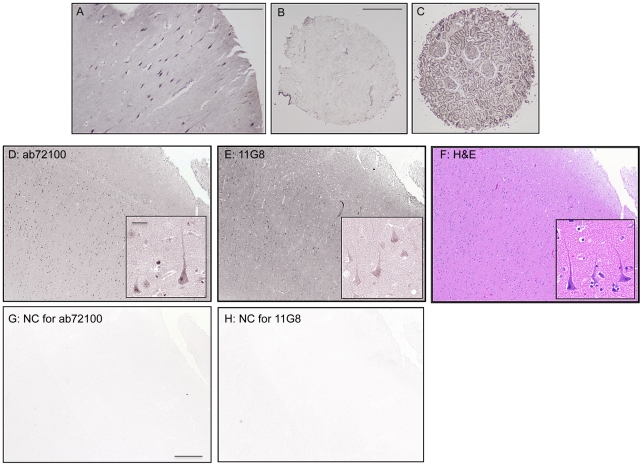
CXCR7 protein expression in the human brain. Staining with 11G8 or ab72100 was performed on different human tissues, using either a tissue array containing samples from multiple normal organs or additional brain sections as described in the methods. Images from the multi-organ array are shown in the top panels (A to C; scale bars: 200 µm). Neuronal CXCR7 expression was observed in the gray matter (A) while no staining is observed in normal breast tissue (B), as expected. CXCR7 was also expressed in the kidney (C). Additionally, serial sections from human frontal cortex were stained with: (D) rabbit polyclonal antibody ab72100, (E) 11G8, or (F) H&E (details in methods). Similar neuronal staining was observed with both CXCR7 antibodies. No staining was detected when the primary antibody was omitted (G) or replaced by the isotype control (H). Images in insets are higher magnification of D, E, and F. Scale bars: 400 µm (D–H), 50 µm for insets. NC = negative control.

Next, a tissue microarray containing samples of various normal human organs, including the brain, was analyzed. Initial studies with 11G8 showed a typical neuronal staining in the cerebral gray matter (Figure 2A/E); CXCR7 staining was also seen in tubular formation in the kidney (Figure 2C), in line with a previous study [37], while normal breast tissue was negative as expected (Figure 2B). Staining in the gray matter was also confirmed by using another CXCR7 antibody (ab72100, Figure 2D) that recognizes distinct portions of the receptor, i.e. epitopes in the second extracellular loop as opposed to the N-terminus recognized by 11G8. Though both these antibodies indicated neuronal expression of CXCR7, we primarily used 11G8 to keep consistency with the rest of the data.

**Figure 3:**
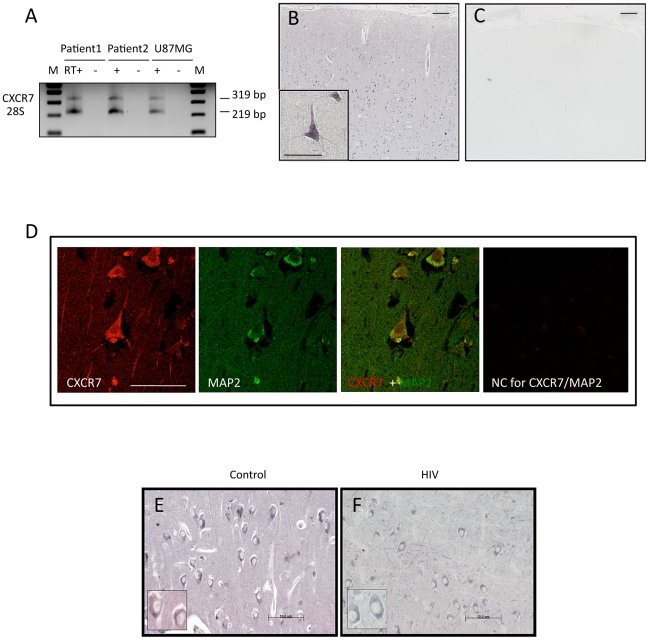
CXCR7 mRNA/protein expression in the brain of normal subjects and HIV patients. (A) CXCR7 mRNA expression in the frontal cortex of control subjects; human glioblastoma cells (U87MG) were used as a positive control; M = molecular markers; RT = reverse transcriptase. (B) CXCR7 protein expression in cortex of control patients. (C) No staining was detected in adjacent slices when the primary Ab was replaced by the isotype control. Scale bar is 0.2 mm in panels B through C and 50 µm in the lower left inset of panel B. (D) Double-staining with MAP2 (green) and CXCR7 (red) in human cortex. Scale bar: 50 µm. NC: negative control (shown here is a staining using CXCR7 isotype control, no MAP2 primary, and addition of all secondaries). (E/F) CXCR7 immunostaining was examined in the hippocampus from control subjects (E) and HIV patients (F). Expression of CXCR7 protein seems predominant in cytoplasm in both groups. Scale bars: 10 µm.

Following this initial validation, we started the analysis of the human tissue samples from the NNTC to determine expression of CXCR7 RNA (Figure 3A) and protein (Figure 3B–F) in both control and HIV patients. Brain samples (frontal cortex and hippocampus) from control subjects and HIV+ patients included a total of 9 subjects of different race, age, and gender as indicated in Table 1. The data in normal brain tissue confirmed our previous observations with the tissue arrays. As shown in Figure 3, both CXCR7 RNA and protein are found in the human brain. CXCR7-expressing cells were identified as neurons based on morphology/localization (Figure 3B) as well as co-staining with the neuronal marker MAP2 (Figure 3D). CXCR7-positive cells were observed in both cortex (Figure 3A–D) and hippocampus (Figure 3E/F) of control and HIV patients. Our analysis primarily focused on MAP2 positive cells in the frontal cortex, since the major goal of this study was to evaluate expression of the CXCR7 protein in mature cortical neurons. These IHC studies showed that the vast majority of MAP2 positive cells are also positive to CXCR7. Specifically, within the population of cells that stained positively for MAP2, the percentage of cells also expressing CXCR7 was 95% and 91%, respectively, in control and HIV tissue (total number of cells analyzed = 125 cells; these included CXCR7+/MAP2+, CXCR7+/MAP2-, and CXCR7-/MAP2+). These data suggest that both excitatory and inhibitory mature neurons express the CXCR7 protein and are in agreement with recent studies in the developing brain showing expression of cxcr7 transcripts in migrating interneurons as well as immature excitatory neurons [38]. Furthermore, in these human brain samples only a minority of CXCR7 positive cells (i.e. 11% and 11.4% respectively control/HIV) did not stain for MAP2. As MAP2 is a marker for mature neurons, this smaller group of cells could include resident/infiltrating immune cells (including glia) and/or neural precursors. Further studies are necessary to fully characterize this CXCR7 positive/MAP2 negative cell population (i.e. to establish cell types, differentiation stages, and leukocytes infiltration) and its potential changes during disease progression in specific brain areas. Analysis of a larger group of samples is underway to achieve this goal and to identify potential differences in the expression of CXCR7 between HIV-positive patients without neurological problems and HIV positive patients affected by neurocognitive impairment. The present study demonstrates that CXCR7 protein is widely expressed in mature neurons of the human frontal cortex and hippocampus under both normal and diseased (i.e. HIV) conditions, thus providing the fundamental basis of future investigations focused on its specific role in brain pathology.

### CXCR7 Expression in Cultured Neurons and its relation to CXCR4

**Figure 4:**
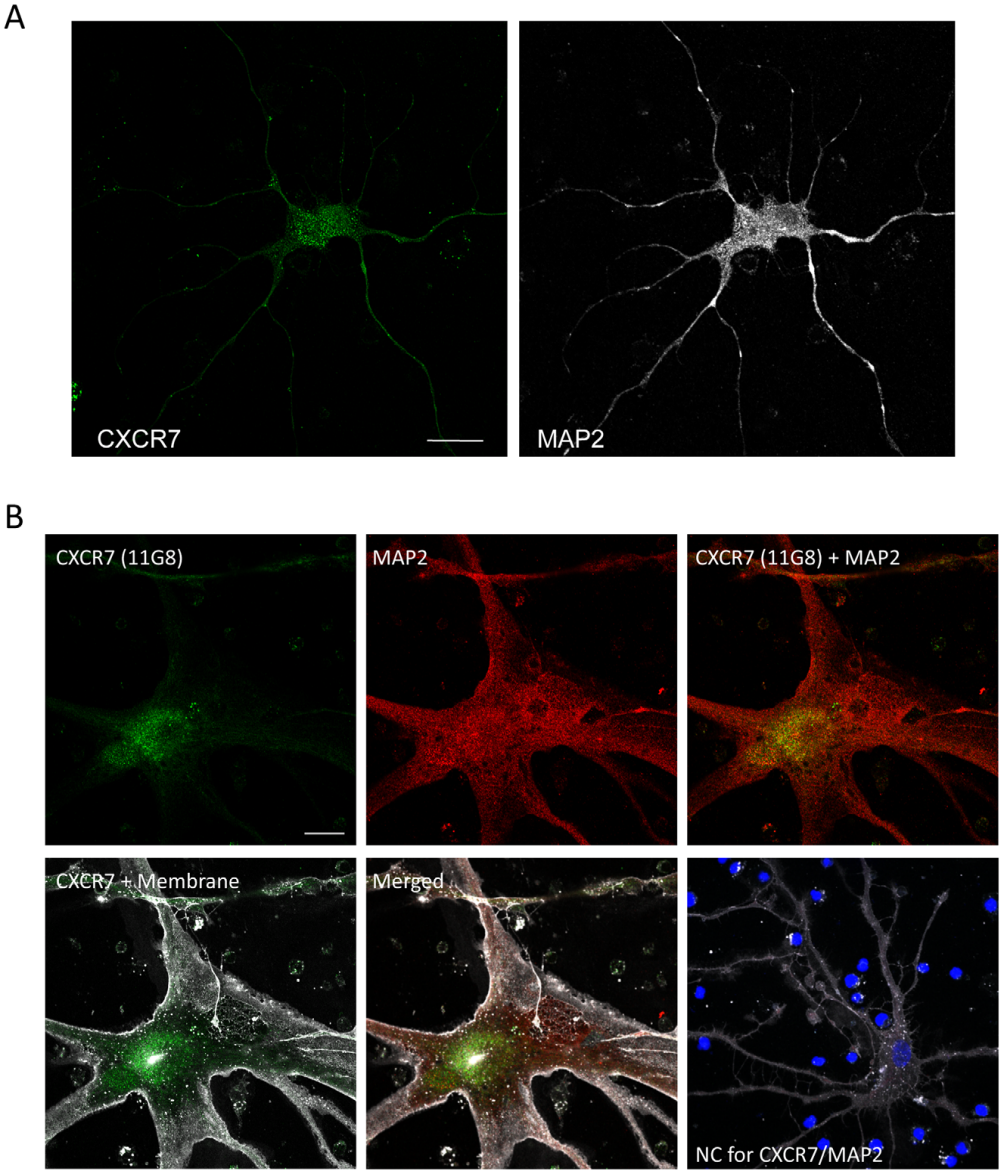
CXCR7 protein expression in human cultured neurons. Human neurons cultured for up to 3 weeks were labeled with a plasma membrane and a nuclear marker prior to fixation as described in the methods, using the Image-iT LIVE Plasma Membrane and Nuclear Labeling Kit. After permeabilization, neurons were stained for chemokine receptors and neuronal marker (MAP2). In neurons, CXCR7 (11G8, green) appears to be localized predominantly to the soma (A), mostly in the perinuclear region (B). A negative control without primary antibodies is shown in lower right panel. Nuclei of surrounding (mostly dead) cells, often found in these primary human cultures, appear in intense blue (i.e. Hoechst staining); membrane staining is shown in white in the bottom row of B. Scale bars: 20 µm.

**Figure 5:**
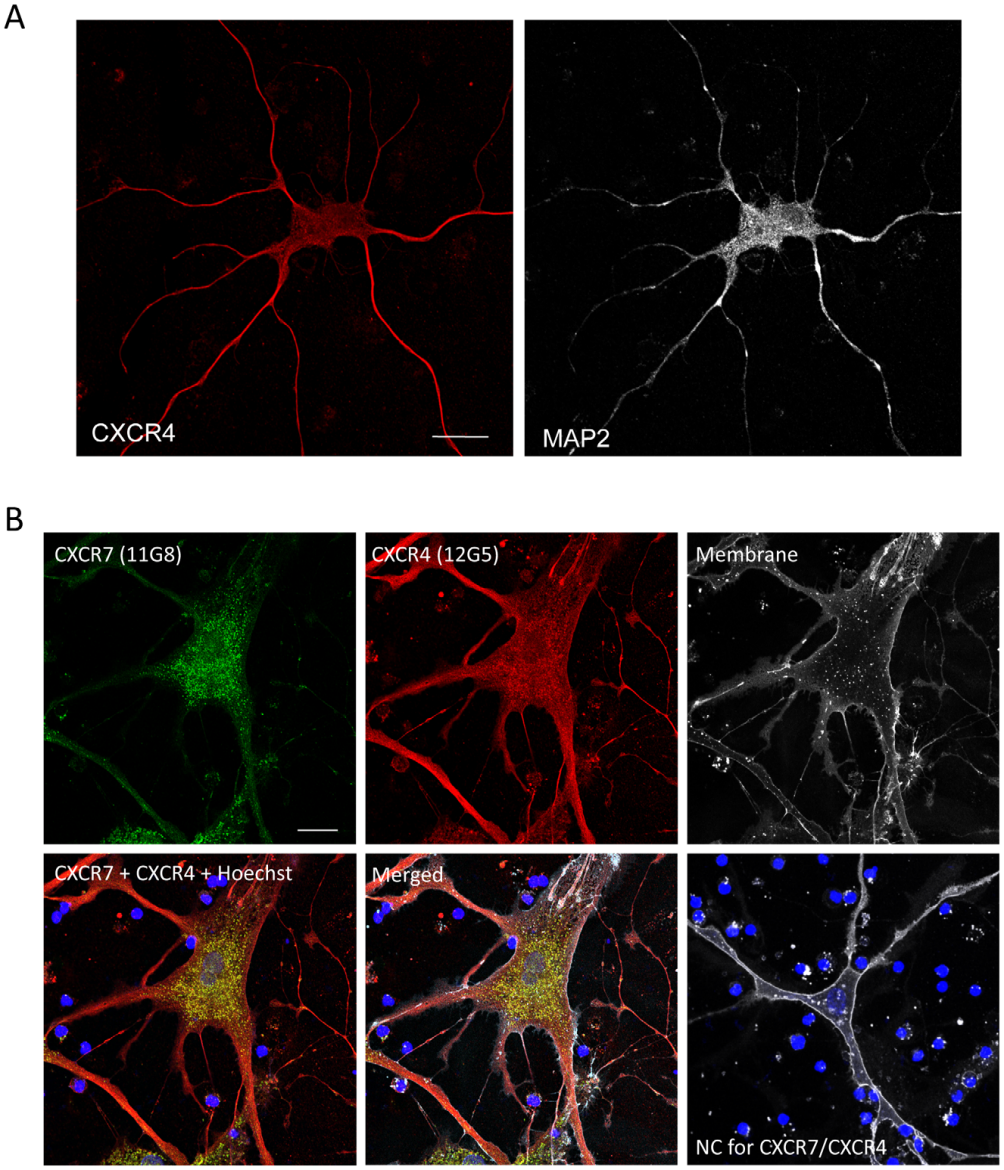
CXCR7 and CXCR4 protein expression in human cultured neurons. Human neurons were labeled with a plasma membrane and a nuclear marker prior to fixation as described previously. After permeabilization, neurons were stained for chemokine receptors and neuronal marker (MAP2). Unlike CXCR7, CXCR4 expression (12G5) in neurons was distributed to both the soma and processes (A) though the intracellular expression of the two receptors overlapped in some areas of the neuronal cell body (B). Negative control (NC, Mouse IgG1 isotype) is shown in lower right panel. Scale bars: 20 µm.

**Figure 6:**
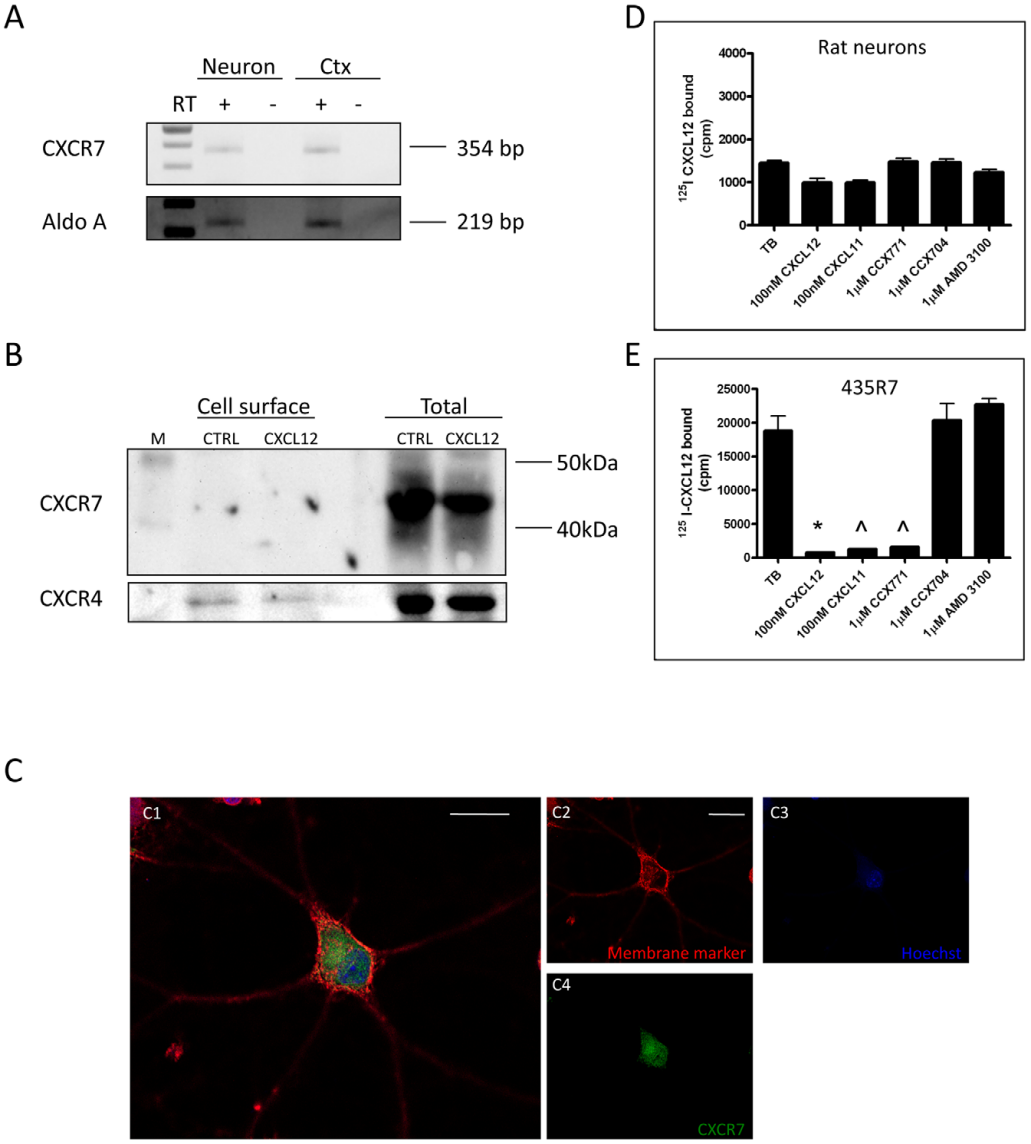
CXCR7 mRNA and protein expression in rat neurons. (A) CXCR7 mRNA was detected in rat primary neurons as well as rat cerebral cortex (Ctx, E17) by RT-PCR as a 354-bp band [33]. (B) Cell surface proteins were purified from 75 µg of control (CTRL) or CXCL12-treated total proteins obtained from pure neuronal cultures. Forty micrograms of total proteins were loaded in the last two lanes. Molecular markers (M) were loaded in the first lane. The same membrane was reprobed with anti-CXCR4. As expected, CXCR4 is expressed on the surface of neurons in control (CTRL), but surface expression is reduced following internalization caused by CXCL12 treatment (1 hr, 20 nM). (C) The subcellular localization of CXCR7 in rat neurons was also examined by confocal microscopy. A membrane (red, C2) and nuclear marker (blue, C3) were applied on live cells prior to permeabilization. Neurons were then stained with anti-CXCR7 mAb (green, C4) and imaged using the Leica TCS SP2 confocal system (Plan Apochromat 63×oil immersion objective, scale bar: 20 µm). All figures are 0.5-μm *z*-scan images. CXCR7 was observed in the cytoplasm, but not on the cell surface, in these primary neurons. (D–E) Binding assays using radiolabeled CXCL12 were used to determine membrane expression of CXCR7 in primary neurons (D). MDA-MB-435 cells transfected with CXCR7 (here labeled as 453R7) were used as positive controls (E). Data represent the mean±SEM. TB, total bound. *p<0.05 vs. TB and vs. AMD3100; ¯*p*<0.05 vs. TB and vs. CCX704.

To gain greater insights about the cellular localization of CXCR7 in neurons, we studied expression of CXCR7 in human brain cultures by immunocytochemistry and confocal microscopy. Most neurons (identified by positive staining for the neuronal marker, MAP2) expressed CXCR7, mainly in perinuclear regions (Figure 4). All CXCR7-positive cells also expressed CXCR4 (Figure 4 and 5A), indicating that CXCR7 and CXCR4 were present in the same populations of neurons. However, CXCR7 was predominantly expressed in the cytoplasm, whereas CXCR4 was also found on the cell membrane. Interestingly, most CXCR7 staining overlaps with CXCR4's cytosolic staining (yellow area in Figure 5B), suggesting that the two receptors may interact at the intracellular level. The phenotype of intracellular retention of CXCR7 did not seem to be limited to neurons but also observed in some non-neuronal human cell lines (i.e. U87MG), as shown in Figure S1. This figure reports studies with different CXCR7 positive (U87MG or U343) and negative (MDA-MB-435) cells. Though CXCR7 transcripts are expressed in both U87MG and U343 cells (Figure S1A) as expected, flow cytometry and radioligand binding assays show CXCR7 protein expression only on the surface of U343 (Figure S1B). Furthermore, CXCR7 was detected in the cytoplasm of U87MG cells by confocal microscopy, but not on the cell surface (Figure S1B). Next, we asked whether this unique pattern of CXCR7 expression is human specific. To this end, we first confirmed the expression of CXCR7 mRNA in cultures of rat cortical neurons as well as in the rat cortex (Figure 6A). Then, we examined the localization of CXCR7 protein in rat neurons using different approaches. Western blot analysis showed no expression of CXCR7 in the surface protein fraction as opposed to the total protein expression (Figure 6B). Surface expression of CXCR4, which is known to internalize after CXCL12 treatment [30], was also probed using the same membrane as an internal control of protein extraction and separation. Immunostaining and confocal microscopy on rat primary neurons also confirmed the lack of CXCR7 protein expression on the neuronal surface (Figure 6C). CXCR7 staining (green) was detected predominantly in the intracellular region and did not co-localize with the membrane marker (red). To further support these results, surface expression of CXCR7 in cultured neurons was assessed by competitive binding studies. In agreement with the Western blot and confocal analysis, radioligand binding failed to demonstrate CXCR7 at the cell surface (Figure 6D/E). Rat neurons cultured for varying periods of time (i.e. up to 14 DIV) were incubated with radiolabeled CXCL12. In contrast to the control (MDA-MB-435 cells transfected with CXCR7, i.e. 435R7), minimal binding of ^125^I-CXCL12 is observed in rat neurons (independently of the age *in vitro*, data not shown), confirming the lack of surface CXCR7. Collectively, these results indicate that surface expression of CXCR7 is negligible in cultured rat/human neurons while a substantial pool of the receptor is found in the intracellular compartment.

### High-Affinity CXCR7 ligands do not alter CXCL12-induced ERK1/2 and Akt activation

**Figure 7:**
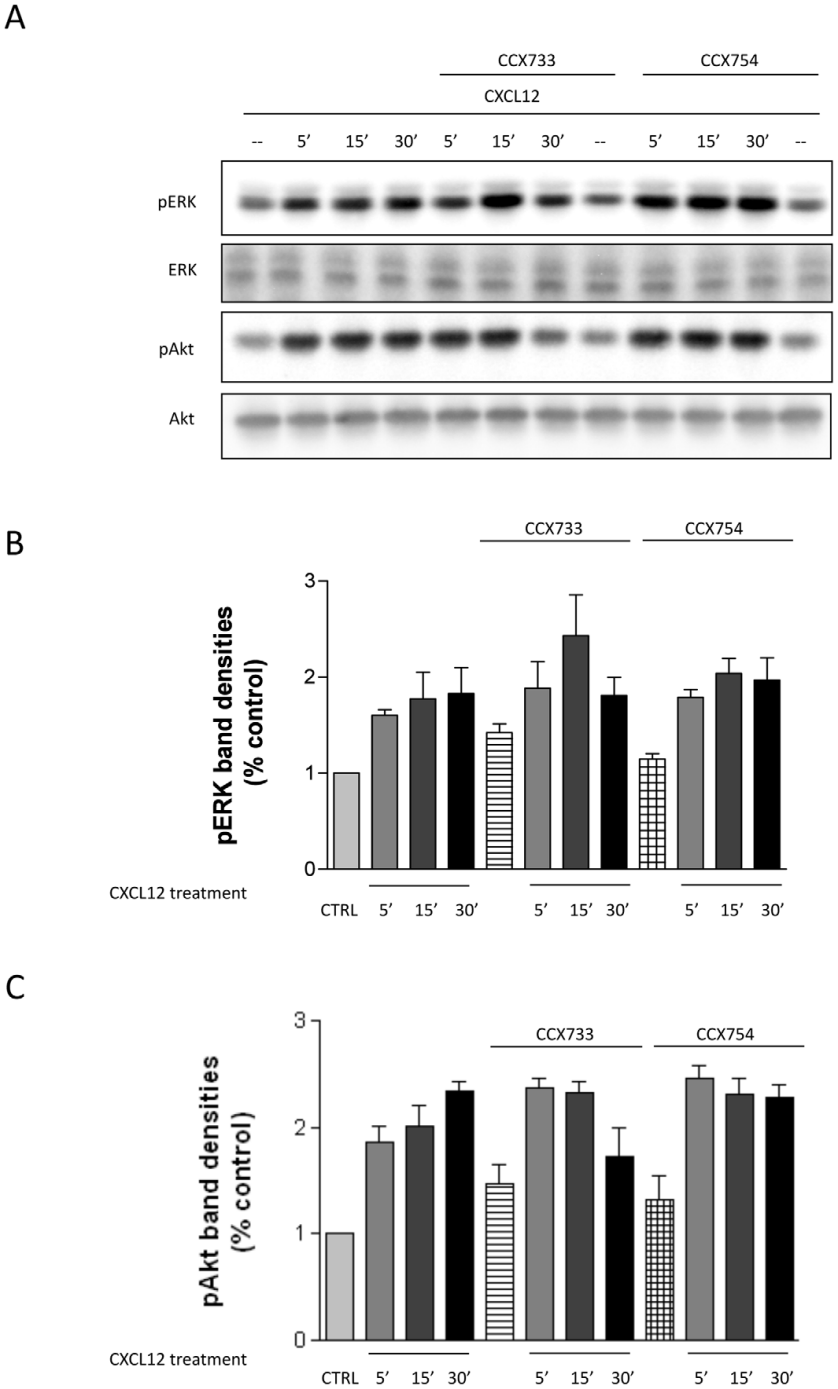
CXCL12-induced phosphorylation of ERK or Akt in the presence of CXCR7 ligands. (A) Rat cortical neurons were treated with the CXCR7 ligands CCX733 (100 nM) or CCX754 (100 nM) 15 minutes before (and during) their exposure to vehicle or CXCL12 (20 nM, as indicated). Activation of survival pathways (i.e., ERK/Akt) induced by CXCL12 was measured by Western blot analysis using phosphospecific antibodies [30]. (B/C) Densitometric analysis showed no significant difference in pERK (analysis of variance [ANOVA]; NS, part B) or pAkt (ANOVA; NS, part C) bands after CCX733/754 treatment compared with CXCL12 alone. Total ERK and Akt were used for normalization of pERK and pAkt, respectively. Data represent the mean±SEM, as obtained in three independent experiments.

The above results suggest limited to null expression of CXCR7 on the neuronal surface. Thus, functional assays were performed to validate these observations with a different approach as well as to establish the potential contribution of even minimal CXCR7 expression to CXCR4 signaling. Rat neurons were exposed to CXCR7-specific ligands (CCX733 or CCX754, 100 nM) known to inhibit CXCL12 binding to CXCR7 [2], [8], [9], [25]. The goal was to determine whether treatment with the small-molecule compounds would affect the activation of intracellular pathways stimulated by CXCL12, specifically phosphorylation of ERK1/2 and Akt. As we previously demonstrated, activation of these pathways in neurons depends on CXCR4, involves Gi/o proteins, and ultimately promotes neuronal survival [30], [35], [39]. The results presented here show that CCX733 or CCX754, applied to pure neuronal cultures 15 minutes before and during CXCL12 treatment (20 nM, for indicated time), did not significantly alter CXCL12-induced stimulation of ERK1/2 and Akt (Figure 7). The effect of 100 nM CCX733/754 is reported here, but similar results were obtained in additional experiments using a wider range of CCX concentrations, i.e. from 10 nM to 1 µM (not shown). Also, the CXCR7 ligands did not induce ERK or Akt phosphorylation (nor Gi stimulation, not shown) in the absence of CXCL12 (not shown). These results are consistent with the lack of CXCR7 expression on the neuronal surface and with previous reports with similar compounds [21].

### CXCR7 Protein Partially Co-localizes with The Endoplasmic Reticulum Marker ERp29 in Human Neurons

**Figure 8:**
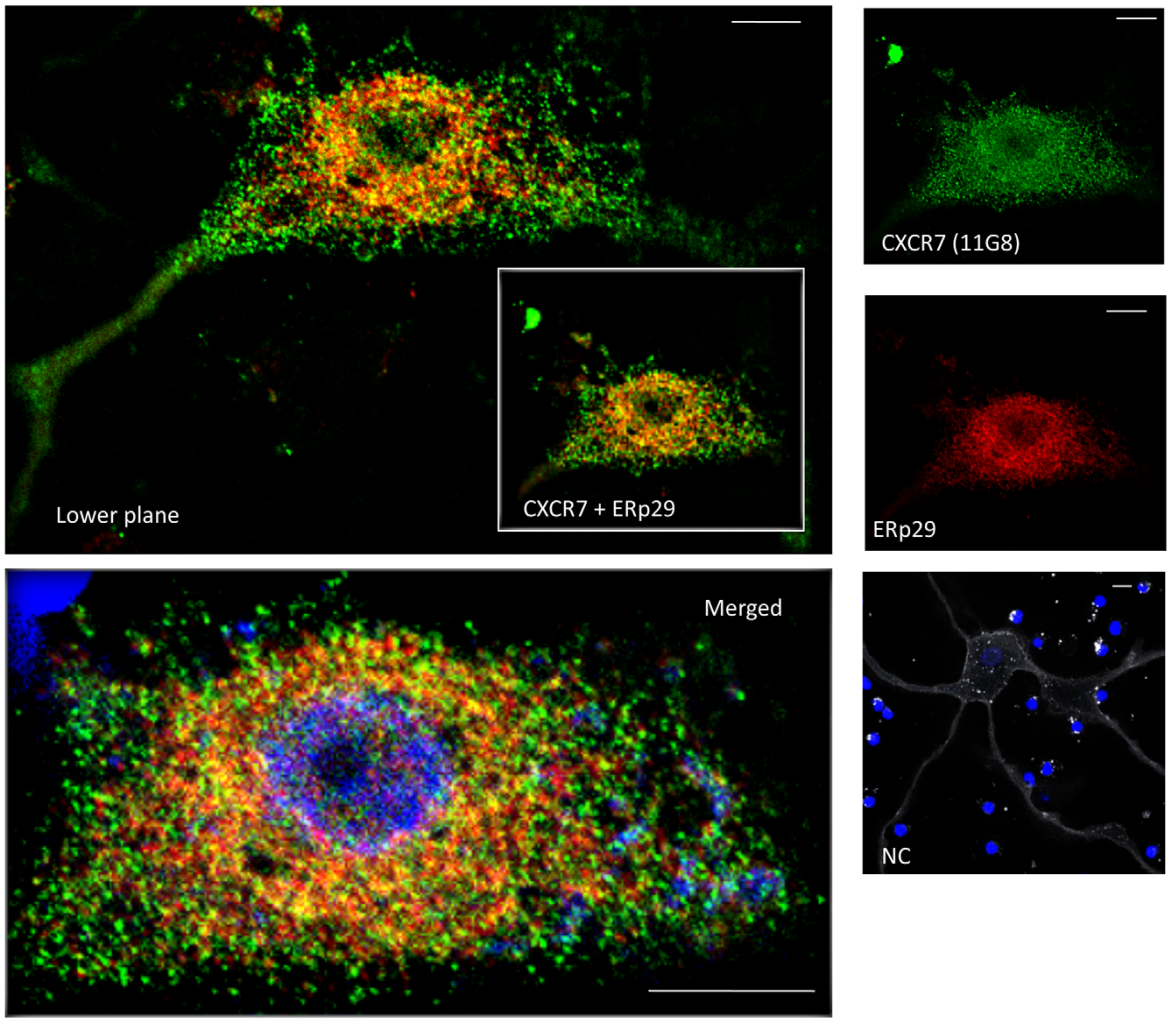
CXCR7 partially co-localizes with endoplasmic reticulum marker ERp29. Human neurons were incubated with antibodies against CXCR7 (11G8, green) and ERp29 (red) as reported above and in the methods. Mouse IgG1 isotype control was used as negative control (NC). Overlapping staining of CXCR7 and ERp29 (yellow) is detected in many neurons mostly in the perinuclear region. Image labeled as “Merged” is an enlargement of the CXCR7+ERp29 image shown in the inset plus the nuclear staining, whereas the image labeled as “lower plane” is the merge of CXCR7+ERp29 from a different plane of the same cell. Scale bar: 10 µm.

The data presented show that CXCR7 is expressed in intracellular compartments in differentiated neurons, but the exact localization is still unclear. Previous studies in human T lymphocytes reported expression of CXCR7 in endosome vesicles, though this finding is still controversial [15]. However, CXCR7 does not co-localize with early (EEA1) or late (Rab7, Rab11) endosome markers in human neurons (Figure S2), suggesting that CXCR7 plays different roles in neurons and immune cells. Further analysis in neurons revealed a partial co-localization of CXCR7 with the endoplasmic reticulum (ER) marker ERp29 (Figure 8) [40]. The ER is part of the secretory pathway implicated in protein translocation to the cell surface, and it is commonly considered a default pathway. These data suggest that CXCR7 may be re-directed to the cell surface under specific cellular conditions or be implicated in trafficking of other proteins. Based on the CXCR7/CXCR4 expression pattern shown previously (Figures 4–5), this may include CXCR4-a hypothesis currently under investigation in our laboratory.

## Discussion

The 7TM receptor CXCR7 has received considerable attention in the last few years, following the discovery of binding to chemokines that are implicated in essential biological functions, such as the chemokine CXCL12 [1], [2]. Initial studies reported expression of CXCR7 transcripts in various organ systems-including the central nervous system [2], [4]-but the actual level of CXCR7 protein, its signaling capability, and related function in different cell types are still not clearly defined. As demonstrated by pioneering studies in rodents lacking CXCL12 or CXCR4 [41], as well as by other evidence *in vitro* and *in vivo*, CXCR4 is the specific and primary CXCL12 receptor. However, one major question concerns the role of CXCR7 in modulating the effect of the CXCL12/CXCR4 axis in the brain and other organs. Though several hypotheses have been proposed (e.g. the scavenger or decoy activity of CXCR7; a CXCR4/CXCR7 signaling cross-talk or physical interactions between the two receptors that may affect signaling) data about the expression of the CXCR7 protein in individual brain cells are still scarce and inconclusive. Furthermore, the limited availability of research reagents that effectively work in multiple animal species has hindered progress in this field [24]. Only very recently studies that primarily looked at expression of cxcr7 transcripts in migrating neurons using different transgenic mouse lines have shed some light on CXCR7/CXCR4 interaction in the developing brain [38], [42].

Here we focused on the protein expression of CXCR7 in human differentiated neurons as a foundation to further investigation on the role of CXCR7 in the neuroprotective action of CXCL12. The results shown here, using different antibodies, experimental approaches, and multiple controls indicate that CXCR7 protein expression in mature neurons is mostly limited to the intracellular compartment and seem to exclude a prominent role of this receptor in the immediate regulation of typical pathways triggered by neuronal CXCR4, such as the activation of ERK1/2 and Akt [30]. This is suggested by the perinuclear localization of the CXCR7 staining in human brain tissue and cultured neurons and the inability of the high-affinity CXCR7 ligands to inhibit CXCL12 binding as well as CXCL12/CXCR4 functional assays in rat neurons. Interestingly, in migrating interneurons CXCR7 can contribute to CXCL12 signaling by regulating surface expression of CXCR4 and, in line with our data, it is prevalently expressed at the intracellular level [38]. Another study [42] suggested that CXCR4 and CXCR7 have distinct functions in the regulation of interneuron movement and laminar positioning. Though both of these studies are in agreement with our findings concerning localization of CXCR7 in differentiated neurons, the distinct roles of CXCR7 in mature neurons and neural precursors (which also express CXCR7 [21]) remain to be established.

Furthermore, disconnect between expression of the CXCR7 message and its expression on the surface of cells is not totally unexpected. While transcription of many 7TM receptors leads to a rapid translation and transport of receptor to its signaling position at the cell surface, some receptors appear to be retained in the cell cytoplasm as a form of post-translation regulation. For instance the chemokine receptor CXCR2 can be sequestered to the cytoplasm of neutrophils, with its surface expression regulated by glycosylation, which serves to release the intracellular pool to the cell membrane [43]. Similarly, N-linked glycosylation appears to play a role in correct folding and trafficking to the plasma membrane of the vasoactive intestinal peptide receptor 1 (VIPR1) and GABA-transporter 1 receptors [44], [45]. Thus, post-translational retention of CXCR7 in the endoplasmic reticulum may play an important role in the biology of the CXCR7 receptor, with post-translational modifications or other external stimuli regulating localization and function.

Moreover, the co-expression of CXCR4 and CXCR7 in specific compartments suggests that CXCR7 may interact with CXCR4 at the intracellular level; for instance, CXCR7 may control surface levels of CXCR4 by limiting its intracellular trafficking (supported by the presence of CXCR7 in the ER) or it can alter CXCR4 interaction with other signaling molecules as suggested in other studies [14]. However, protein expression and localization of CXCR7 seems to be specifically regulated in different cell types and its function may significantly vary in neuronal versus non-neuronal cells. This is suggested by studies in lymphocytes and breast cancer cells showing CXCR7 protein expression in different intracellular compartments [8], [15], such as early/late endosomes and lysosomes. Interestingly, studies on other 7TM receptors, such as the delta opioid receptor [46], the D1 receptor [47], [48], and the CCR5 chemokine receptor [49], have shown that these receptors normally reside in the cytoplasm and translocate to the cell membrane upon interaction with chaperones/escort proteins triggered by specific stimuli (see review [50]). Other studies have reported that in T lymphocytes the majority of CCR5 is found in the ER and the Golgi apparatus [49] but is transported to the cell surface after transfection of CD4, indicating that receptor surface expression is regulated by specific factors [49]. TNF-α and IL-1β can up-regulate chemokine receptors (including CXCR7) in different cells [2], [21]. However, we did not find this to be the case in human/rat neurons, as CXCR7 expression is still negligible on the surface of human neurons treated with TNF-α or IL-1β (10 ng/mL, up to 72 hrs; data not shown). As alterations of CXCL12/CXCR4 function are thought to contribute to neuropathology, including neuroAIDS, we also asked whether changes in CXCR7 expression between normal and HIV-positive brain tissue could be involved in CXCR4 impairment. A first step in this direction was to examine overall expression of the CXCR7 protein in a few representative brain areas from control and HIV subjects in order to gather preliminary information about distribution of CXCR7 in the adult brain under physiological and pathological conditions. Based on the information collected from these studies, i.e. general distribution of CXCR7 in the two group of subjects, control and HIV-positive, we have started a larger and more detailed analysis of CXCR7 expression as it relates to neurocognitive impairment by using multispectral microscopy and laser capture microdissection. Though progress of these studies is relatively slow, due to the complexity of the technical approach and limited availability of the brain samples, they will provide crucial information about the role of CXCR7 in the regulation of CXCR4 *in vivo*.

In conclusion, our data provide the first evidence of CXCR7 protein expression in human differentiated neurons, both *in vivo* and in culture, laying the foundation for subsequent studies to characterize the physiological and pathological role of CXCR7 in the adult brain. Collectively, the results so far exclude a substantial role of CXCR7 in the short-term survival signaling stimulated by CXCL12 in neurons. This is not only an important information about the overall function of CXCL12 in the brain, but a potential positive result supporting ongoing efforts toward development of CXCR7-based therapies against cancer and other pathologies. Based on these data, use of specific CXCR7 ligands that target CXCR7-positive cells [12], [27] is not expected to be associated with direct neuronal damage or immediate impairment of neuronal CXCR4 function. However, these studies also raise the possibility that CXCR7/CXCR4 interactions in intracellular compartments can control CXCR4 trafficking to the surface and regulate CXCR4 coupling to other proteins. As recycling/degradation and delayed intracellular signaling are essential components of 7TM receptors' physiopathology, this supportive action of CXCR7 may have important consequences on CXCR4 long-term function. In line with these conclusions, a recent report has shown that CXCR7 can control CXCR4 levels *in vivo* via modulation of its degradation [38]. Therefore, further characterization of this interesting protein in specific brain cell populations under normal and pathological conditions should lead to a better understanding of the numerous CXCL12 functions in the CNS and should be highly encouraged.

## Supporting Information

Figure S1 **Variable expression of CXCR7 protein on the cell surface in different CXCR7-positive cells.** CXCR7 mRNA was detected in U87MG and U343 cells, but not the MB-MDA-435 or no template controls by RT-PCR. Despite abundant CXCR7 message (A), cell surface CXCR7 could not be demonstrated in U87MG cells by radioligand binding (B, top) or FACS (B, bottom) in contrast to the U343 positive controls. Using immunofluorescence, CXCR7 was localized to the cytoplasm of U87MG cells (C1) as reported in neurons (C2/C3). Data represent the mean±SEM. Scale bar: 20 µm.
(TIF)

Figure S2 **CXCR7 does not colocalize with early to late endosome markers.** Human neurons were stained for CXCR7 and early (EEA1) or late (Rab7, Rab11) endosomal markers. CXCR7 staining does not overlap with any of the markers used in this study. Mouse IgG1 isotype control was used as negative control (NC). Scale bars: 20 µm.
(TIF)
