# Supplementary figures and images for: CXCR7 Protein Expression in Human Adult Brain and Differentiated Neurons
Source: PLoS One. 2011 May 31;6(5):e20680. doi: 10.1371/journal.pone.0020680 (PMC3105114; doi:10.1371/journal.pone.0020680)

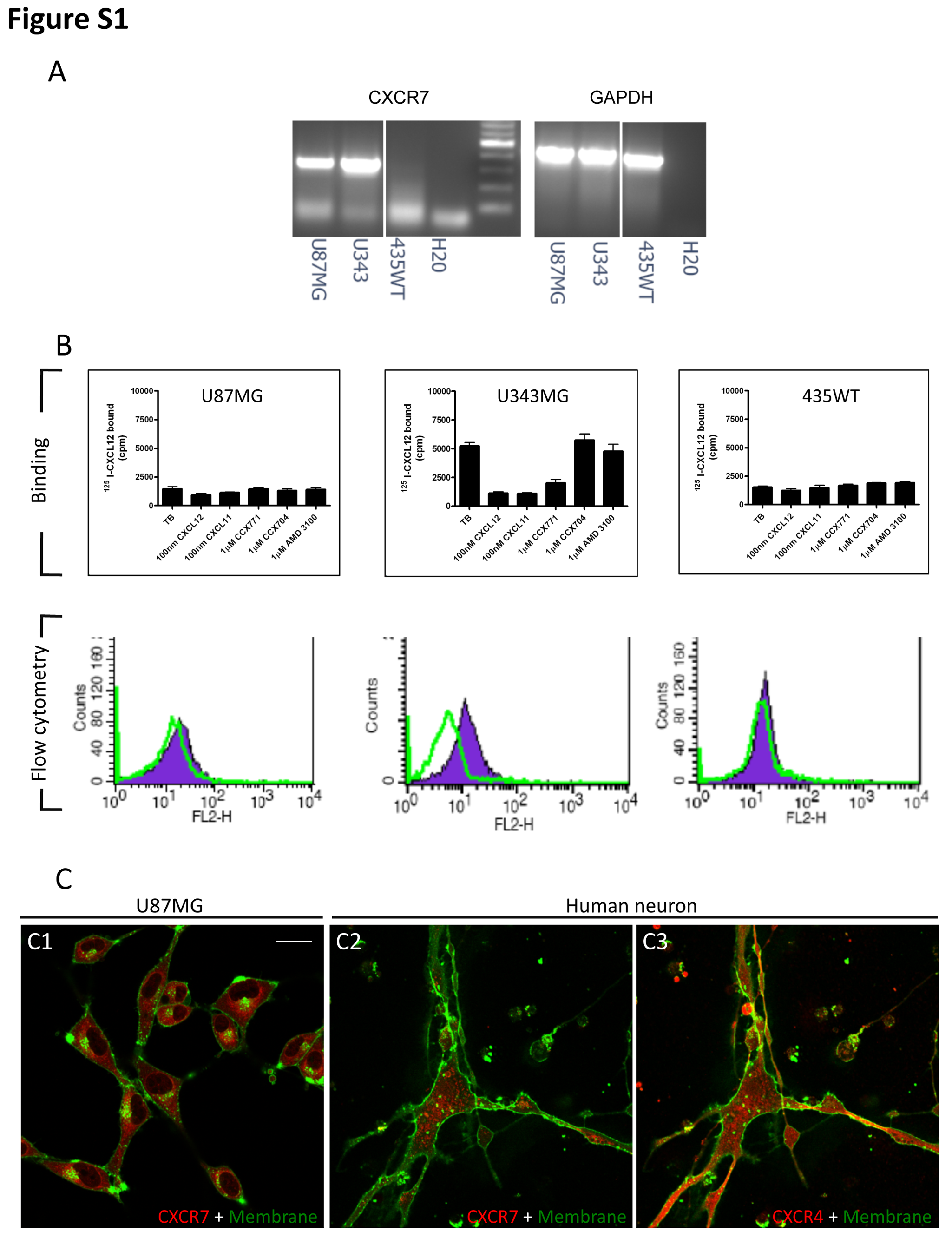

Supplement: Figure S1 — Variable expression of CXCR7 protein on the cell surface in different CXCR7-positive cells. CXCR7 mRNA was detected in U87MG and U343 cells, but not the MB-MDA-435 or no template controls by RT-PCR. Despite abundant CXCR7 message (A), cell surface CXCR7 could not be demonstrated in U87MG cells by radioligand binding (B, top) or FACS (B, bottom) in contrast to the U343 positive controls. Using immunofluorescence, CXCR7 was localized to the cytoplasm of U87MG cells (C1) as reported in neurons (C2/C3). Data represent the mean±SEM. Scale bar: 20 µm. (TIF) [file pone.0020680.s001.tif]

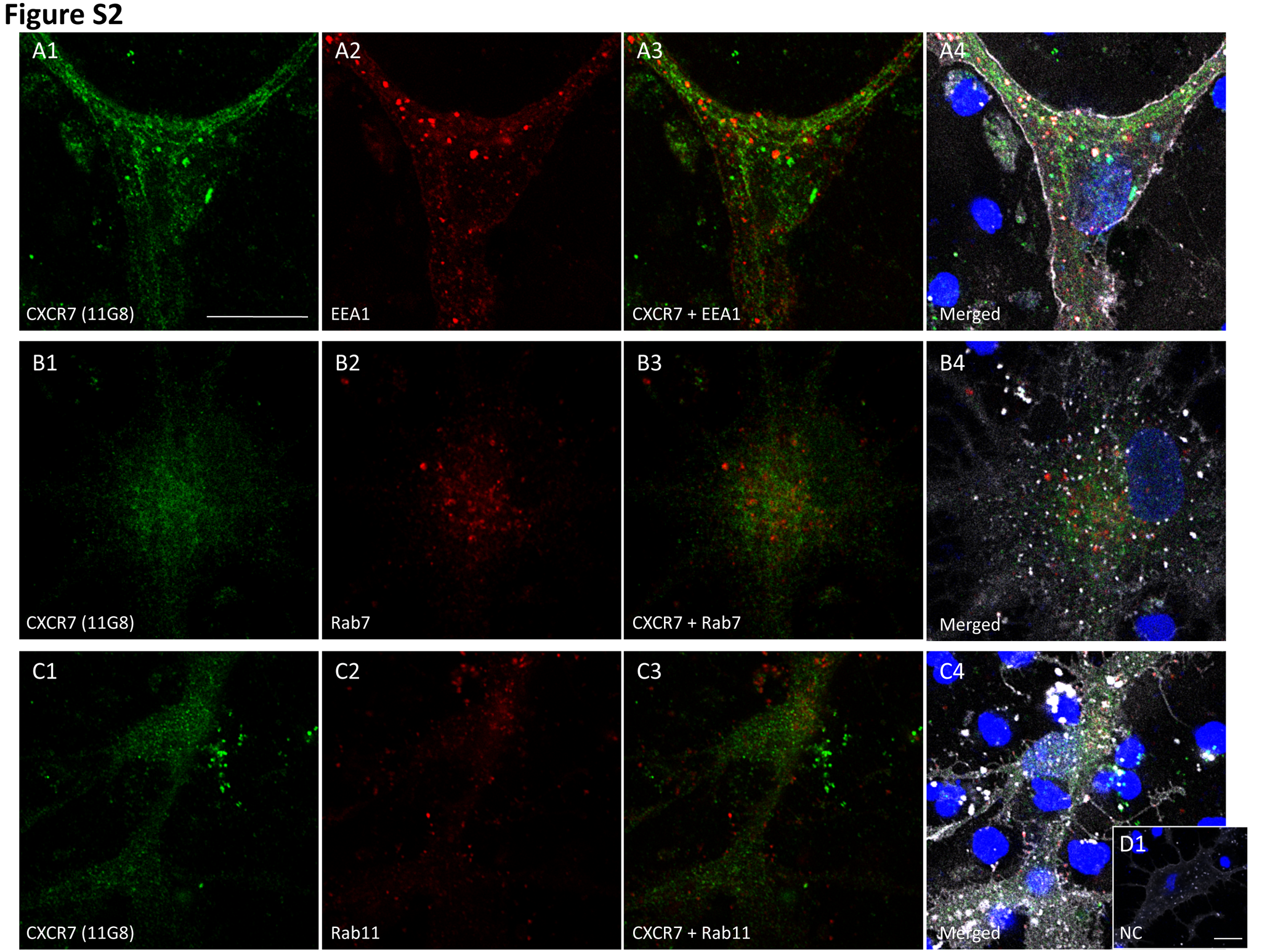

Supplement: Figure S2 — CXCR7 does not colocalize with early to late endosome markers. Human neurons were stained for CXCR7 and early (EEA1) or late (Rab7, Rab11) endosomal markers. CXCR7 staining does not overlap with any of the markers used in this study. Mouse IgG1 isotype control was used as negative control (NC). Scale bars: 20 µm. (TIF) [file pone.0020680.s002.tif]
